# The Renin–Angiotensin System Modulates Dopaminergic Neurotransmission: A New Player on the Scene

**DOI:** 10.3389/fnsyn.2021.638519

**Published:** 2021-04-22

**Authors:** Tamara Kobiec, Matilde Otero-Losada, Guenson Chevalier, Lucas Udovin, Sofía Bordet, Camila Menéndez-Maissonave, Francisco Capani, Santiago Pérez-Lloret

**Affiliations:** ^1^Centro de Altos Estudios en Ciencias Humanas y de la Salud, Universidad Abierta Interamericana, Consejo Nacional de Investigaciones Científicas y Técnicas, Buenos Aires, Argentina; ^2^Centro de Investigaciones en Psicología y Psicopedagogía, Facultad de Psicología y Psicopedagogía, Universidad Católica Argentina, Buenos Aires, Argentina; ^3^Facultad de Psicología y Psicopedagogía, Pontificia Universidad Católica Argentina, Buenos Aires, Argentina; ^4^Departamento de Biología, Universidad Argentina John F. Kennedy, Buenos Aires, Argentina; ^5^Facultad de Ciencias de la Salud, Universidad Autónoma de Chile, Santiago de Chile, Chile; ^6^Facultad de Medicina, Pontificia Universidad Católica Argentina, Buenos Aires, Argentina; ^7^Departamento de Fisiología, Facultad de Medicina, Universidad de Buenos Aires, Buenos Aires, Argentina

**Keywords:** Parkinson’s disease, renin–angiotensin system, dopaminergic synapse, nigrostriatal circuit, dopaminergic neurotransmission

## Abstract

Parkinson’s disease (PD) is an extrapyramidal disorder characterized by neuronal degeneration in several regions of the peripheral and central nervous systems. It is the second most frequent neurodegenerative disease after Alzheimer’s. It has become a major health problem, affecting 1% of the world population over 60 years old and 3% of people beyond 80 years. The main histological findings are intracellular Lewy bodies composed of misfolded α-synuclein protein aggregates and loss of dopaminergic neurons in the central nervous system. Neuroinflammation, apoptosis, mitochondrial dysfunction, altered calcium homeostasis, abnormal protein degradation, and synaptic pathobiology have been put forward as mechanisms leading to cell death, α-synuclein deposition, or both. A progressive loss of dopaminergic neurons in the substantia nigra late in the neurodegeneration leads to developing motor symptoms like bradykinesia, tremor, and rigidity. The renin–angiotensin system (RAS), which is involved in regulating blood pressure and body fluid balance, also plays other important functions in the brain. The RAS is involved in the autocrine and paracrine regulation of the nigrostriatal dopaminergic synapses. Dopamine depletion, as in PD, increases angiotensin II expression, which stimulates or inhibits dopamine synthesis and is released via AT1 or AT2 receptors. Furthermore, angiotensin II AT1 receptors inhibit D1 receptor activation allosterically. Therefore, the RAS may have an important modulating role in the flow of information from the brain cortex to the basal ganglia. High angiotensin II levels might even aggravate neurodegeneration, activating the nicotinamide adenine dinucleotide phosphate (NADPH) oxidase complex, which leads to increased reactive oxygen species production.

## Introduction

Parkinson’s disease (PD) is a complex neurodegenerative disease closely tied to genetic and environmental factors (Simon et al., 2020). This form of extrapyramidal disorder affects movement regulation and emotional management. The progressive loss of dopaminergic neurons in the substantia nigra (SN) pars compacta (SNpc) leads to striatal dopamine (DA) depletion and disrupted motor control. Also, the prefrontal cortex, anterior cingulate gyrus, and frontostriatal pathways are affected by this disease (Blesa et al., 2017; Maiti et al., 2017; Bhat et al., 2018).

Following Alzheimer’s disease, PD is the second most frequent neurodegenerative disease (Delamarre and Meissner, 2017; Cho et al., 2019). Its estimated prevalence varies according to ethnicity, sex, geographic location, phenotype, and environment, and rounds 5–35 cases per 100,000 individuals worldwide (Cho et al., 2019; Simon et al., 2020). It is a rare condition before the age of 50 years (Bhat et al., 2018), but its incidence increases 5- to 10-fold from 60 to 90 years old. In fact, the estimated global incidence of 0.3% reaches 1% in people beyond their 60s and 3% in those over their 80s (Poewe et al., 2017; Angelopoulou et al., 2020). The world population is aging. As PD incidence depends on age, it is expected to rise in the coming years. The proportion of people over 60 years old might double by 2050, and so PD incidence (Cho et al., 2019; Angelopoulou et al., 2020). Thus, this disease is considered an important public health problem, with high personal, social, and economic burdens. Mortality does not increase over the first decade after PD onset and doubles later in the general population (Lee and Gilbert, 2016; Cascabelos, 2017). In the last decades, medical advances have contributed to increasing life span in PD patients, with the expected increase in the mentioned burdens and a rising need for effective therapeutic treatments (De Virgilio et al., 2016; Delamarre and Meissner, 2017).

Parkinson’s disease presents with motor and non-motor symptoms. Among the former, slowness of movement (bradykinesia), postural instability, resting tremor, and muscular rigidity are the most frequent. Non-motor manifestations are dysautonomia, sleeping disorder, cognitive impairment, olfactory dysfunction, and depression and often appear before the onset of the classic motor symptoms (De Virgilio et al., 2016; Maiti et al., 2017; Bhat et al., 2018). Cardinal motor features are evident only after 60–80% of dopaminergic neurons are lost and striatal DA concentration falls below 70–90%, owing to the work of earlier compensatory mechanisms. The central nervous system (CNS) cell loss and striatal denervation increase exponentially with the disease progress, and exacerbated symptoms may turn into a source of disability (Elbaz et al., 2016; Blesa et al., 2017).

## Parkinson’s Pathophysiology: A Deleterious Chain Reaction

The main PD pathologic hallmarks include the abnormal deposition of α-synuclein in intracytoplasmic and intraneuritic inclusions known as Lewy bodies (LBs) and Lewy neurites (LNs) in several brain regions (Elbaz et al., 2016; Simon et al., 2020). Although PD exact etiology remains unclear, a complex interplay among α-synuclein deposition, oxidative stress, mitochondrial dysfunction, neuroinflammation, excitotoxicity, impaired autophagy, and genetic mutations is of consensus (Maiti et al., 2017; Poewe et al., 2017; Angelopoulou et al., 2020). PD involves many genetic–molecular entities affecting different systems, manifesting in a broad spectrum of motor and non-motor features. The most important histological finding is the intracellular LBs, composed of misfolded α-synuclein aggregates. Mitochondrial dysfunction, apoptosis, neuroinflammation, inadequate protein degradation, altered calcium homeostasis, and synaptic pathobiology are mechanisms that result in cell death and/or α-synuclein deposition (Lang and Espay, 2018).

Braak and colleagues () have proposed six stages in PD development. According to their explanation, the α-synucleinopathy of the brain spreads in a caudorostrally ascending and orchestrated way in a bottom-up disease progression. In the pre-symptomatic PD phases (stages 1 and 2), LBs and LNs are only in the olfactory bulb, enteric plexus, and motor branch of cranial nerve X, spreading then to the medulla oblongata, the pontine tegmentum, and the locus coeruleus. In stages 3 and 4, the SN, amygdala, pedunculopontine tegmental nucleus, Meynert nucleus, and temporal cortex are affected, causing clinical symptoms. During stages 5 and 6, sensory association areas and the prefrontal cortex become affected, broadening and deepening PD features, notably those that are cognitive. However, these stages have been supplemented by a theory that suggests that the neocortex is not necessarily the final stage of a bottom-up neurodegenerative process but might be a starting top-down contributor to PD disease (Foffani and Obeso, 2018). Corticostriatal activity may represent a critical stressor for nigrostriatal terminals, promoting secretion of striatal extracellular α-synuclein and its accumulation at dopaminergic synapses, which causes retrograde nigrostriatal degeneration, PD focal motor onset, and progression. Bottom-up and top-down mechanisms could coexist, partly explaining the complex evolution of PD (Foffani and Obeso, 2018). This hypothesis assumes that a dopaminergic nigrostriatal synaptopathy may develop prior to the overt neurodegenerative process. Synaptic vesicle defects and oxidation caused by DA have been mentioned as factors contributing to the vulnerability of the nigral neurons (Wong et al., 2019). Recently, a low level of the synaptic vesicle protein 2A has been reported early in PD patients (Delva et al., 2020), reinforcing the importance of the synaptopathy.

Aging, a striking risk factor for PD, entails changes in this dopaminergic pathway as well (Labandeira-García et al., 2014). The inhibition of the lysosomal autophagy system and/or of the ubiquitin-proteasome system might cause an increase in α-synuclein. Both systems operate to a lesser extent in physiological aging. Besides, mutations associated with genetic PD forms, around 5–10% of total PD prevalence, are related to decreased lysosomal autophagy activity (Poewe et al., 2017). Mitochondrial α-synuclein accumulation affects lysosomal autophagy activity, resulting in oxidative stress; and, conversely, lysosomal autophagy dysfunction leads to α-synuclein accumulation. This vicious cycle increases the reactive oxygen species (ROS) level and decreases adenosine triphosphate (ATP) production, causing neuronal loss and a bioenergetic crisis (Maiti et al., 2017; Poewe et al., 2017; Przedborski, 2017; Surmeier, 2018). In addition, α-synuclein accumulation can cut down synaptic protein levels, impairing neuronal excitability and connectivity (Przedborski, 2017).

The factors that make dopaminergic neurons more vulnerable to degeneration are controversial (Maiti et al., 2017). Dopaminergic neurons in the SNpc might be vulnerable to mitochondrial dysfunction and oxidative stress. Four related reasons may be accountable: (1) the long unmyelinated, or partially myelinated, axons having many transmitter release sites that need a huge energy supply; (2) the autonomous pacemaking activity involves a large calcium influx into the neuron, disturbs calcium homeostasis, and consumes lots of energy as well; (3) the increase in cytosolic DA and its metabolites can lead to oxidative stress; and (4) mitochondrial dysfunction and oxidative stress can cause lysosomal depletion and impaired lysosomal autophagy activity (Michel et al., 2016; Poewe et al., 2017; Lang and Espay, 2018; Surmeier, 2018; Nguyen et al., 2019).

Different neurotransmitter systems in the CNS are involved in PD pathophysiology. Glutamate plays a central role in hindering basal ganglia function. Glutamatergic receptors are involved in neuronal excitability modulation, long-term synaptic plasticity, neurotransmitter release, and definitely in PD neurotransmission impairment (Surmeier, 2018). The *N*-methyl-D-aspartate (NMDA) receptors, a subtype of GLU receptor, are widely expressed in the basal ganglia and excitatory on neurons in the corpus striatum, subthalamic nucleus, globus pallidus, and SN (Zhang et al., 2019). High permeability to calcium and ability to trigger a downstream calcium-dependent signal-transduction cascade of physiological and pathophysiological relevance shape NMDA receptors as key regulators in excitatory synaptic transmission. Glutamate-induced neuronal degeneration might be mediated by NMDA receptors and exacerbated calcium influx (Zhang et al., 2019). Also, cytosolic calcium accumulation in dopaminergic neurons could be related to the sustained engagement of NMDA receptors due to glutamatergic input overactivity in the subthalamic nucleus. Mitochondrial impairment could increase dopaminergic sensitivity to GLU excitotoxicity (Michel et al., 2016). With symptom onset, glutamatergic neurons in the subthalamic and pedunculopontine nuclei that innervate dopaminergic neurons in the SNpc increase glutamatergic firing in the SNpc to compensate for dopaminergic neurons deficit, leading to excitotoxicity (Surmeier, 2018). Overactivation of glutamatergic receptors in corticostriatal, striatopallidal, and subthalamonigral circuitries contributes to neurodegeneration in PD (Duty, 2010).

## General Organization of the Renin–Angiotensin System

**FIGURE 1:**
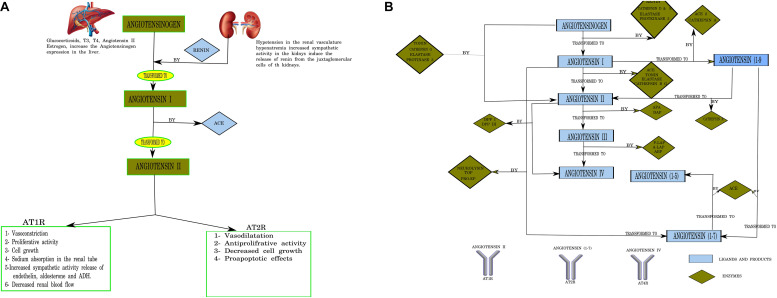
**(A)** Canonical pathways of the renin–angiotensin system (RAS). T3, triiodothyronine; 612 T4, tetraiodothyronine (thyroxine); Angiotensin I (10 AA), Angiotensin I (10 amino acids); Angiotensin II (8 AA), Angiotensin II (8 amino acids); ACE, angiotensin-converting enzyme. Designed using Lucidchart software. **(B)** Pathways of angiotensin peptide formation in the brain. ACE, angiotensin-converting enzyme; ACE2, human homolog of angiotensin-converting enzyme; APA, aminopeptidase A; A-LAP, adipocyte derived leucine-aminopeptidase; TOP, thimet endopeptidase; Pro-EP, prolyl-endopeptidase; DAP, aspartyl aminopeptidase; P-LAP/IRAP, placental leucine-aminopeptidase/insulin-regulated aminopeptidase; APB, aminopeptidase B; DPP IV, dipeptidyl peptidase I; DPP III, dipeptidyl peptidase III; ADH, antidiuretic hormone (vasopresin); aa, amino acids. Numbering of amino acid residues in all fragments is based on the numbering in angiotensinogen. Larger-sized arrows indicate the classical metabolic pathways (Karamyan and Speth, 2007). Designed using Lucidchart software.

The renin–angiotensin system (RAS) is now considered relevant not only in cardiovascular disease but also in physiologic and pathophysiologic mechanisms in different issues and conditions, including neurological diseases (Abadir et al., 2012). It is not only an extracellular canonical system as considered in the past but can be synthesized and used inside the cell as well (Abadir et al., 2012). The CNS, among others, has intracellular binding RAS component receptors (Jackson et al., 2018). The canonical RAS comprises a two-step enzymatic cascade catalyzed by renin and the angiotensin-converting enzyme (ACE) to generate Ang I, II, III, and IV. The major RAS effector peptide, Ang II, has two binding receptor subtypes, type 1 (AT1R) and type 2 (AT2R; Unger et al., 1988; Figure 1). The peptidase activity of renin breaks the Leu–Val bond in angiotensinogen, producing the decapeptide angiotensin I. Sympathetic renal activity, a decrease in intrarenal blood pressure below 90 mmHg of systolic blood pressure in juxtaglomerular cells, or a decrease in sodium and chloride supply to the dense macula cells stimulate juxtaglomerular cells’ renin production.

Circulating Ang II is generated from Ang I by ACE carboxypeptidase activity in the pulmonary endothelial cells (Herichova and Szantoova, 2013; Su et al., 2015), and its effects are mediated mainly via AT1R receptors. The RAS axis is of scientific, medical, and pharmacological interest owing to its effects on blood pressure and heart hypertrophy (Herichova and Szantoova, 2013) (Figure 1).

While traditional and canonical functions of the RAS in the brain have been established, the recognition of multiple Ang receptor subtypes in the brain and emerging functions for Ang-7 and Ang-8 metabolites has expanded the activities attributed to the brain RAS (Wright and Harding, 1994; Saavedra, 2005). Furthermore, other peptides of the RAS process are also expressed in the CNS. Angiotensinogen can be cleaved into angiotensin I by cathepsin D and E, elastase, and proteinase 3. Angiotensin I is cleaved by cathepsin B, tonin, elastase, and proteinase 3 (Rice et al., 2004). Alternatively, angiotensinogen can be directly cleaved to Ang II by cathepsin G, tonin, elastase, and proteinase 3. Angiotensin II is transformed into Ang (Lee and Gilbert, 2016; Blesa et al., 2017; Delamarre and Meissner, 2017; Maiti et al., 2017; Poewe et al., 2017; Bhat et al., 2018; Cho et al., 2019; Angelopoulou et al., 2020; Simon et al., 2020) by cathepsin A and ACE2 and renders Ang (Blesa et al., 2017; Delamarre and Meissner, 2017; Maiti et al., 2017; Poewe et al., 2017; Bhat et al., 2018; Cho et al., 2019; Simon et al., 2020) via cleavage by ACE (Vickers et al., 2002; Figure 1B).

Brain RAS not only regulates blood pressure but also modulates other central functions, including sensory information processing, learning and memory, and emotional responses (von Bohlen und Halbach and Albrecht, 2006). Ang-(Blesa et al., 2017; Delamarre and Meissner, 2017; Maiti et al., 2017; Poewe et al., 2017; Bhat et al., 2018; Cho et al., 2019; Simon et al., 2020) mediates its antihypertensive effects, stimulating synthesis and release of vasodilator prostaglandins and nitric oxide and potentiating bradykinin hypotensive effects (Gulati and Lall, 1996).

Ang II binds AT1R and AT2R, two highly specific receptors. Neuronal AT1R mediates the stimulatory actions of Ang II on blood pressure, water and salt intake, and vasopressin secretion. In contrast, neuronal AT2R stimulates apoptosis and antagonizes AT1R activity (von Bohlen und Halbach and Albrecht, 2006). Many works consider the AT2R as the AT1R counterpart (Guimond and Gallo-Payet, 2012; Garrido-Gil et al., 2013), while others suggest that MasR binding to Ang (Blesa et al., 2017; Delamarre and Meissner, 2017; Maiti et al., 2017; Poewe et al., 2017; Bhat et al., 2018; Cho et al., 2019; Simon et al., 2020) might also antagonize AT1R. The localization of specific brain regions expressing AT1 and AT2 receptors has been reported in humans and mice. Regions related to autonomic function, cognition, and movement control (Guimond and Gallo-Payet, 2012) have been described.

More recently, AT1 and AT2 receptors were found in the basal ganglia, in the SNpc specifically, participating in the DA signaling pathway (Quinlan and Phillips, 1981; Allen et al., 1992). The AT1 and AT2 receptors were identified on neuronal membrane and not in astrocytes in the SNpc (Gendron et al., 2003), and also in the cytoplasm, the nucleus, and mitochondrial membrane (Valenzuela et al., 2016; Perez-Lloret et al., 2017).

### Expression of the Renin–Angiotensin System in the Brain

**FIGURE 2:**
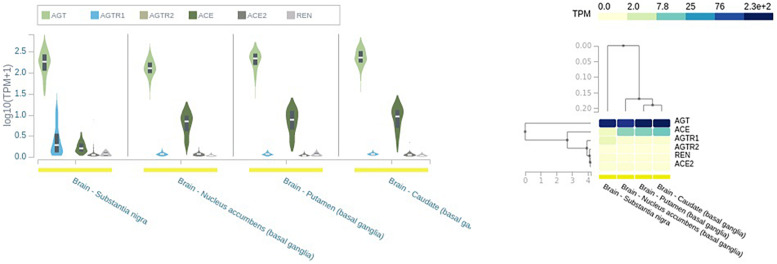
Expression of renin–angiotensin system (RAS) in different brain tissues. AGT, angiotensinogen; REN, renin; ACE, angiotensin-converting enzyme; ACE2, angiotensin-converting enzyme 2; AGTR1, angiotensin type I receptor; AGTR2, angiotensin type 2 receptor; TPM, transcript per millions. Source: https://www.gtexportal.org/home/.

The main angiotensinogen pathway connects the hypothalamus and medulla and is the primary contributor of locally synthesized Ang (Wright and Harding, 2013). In addition, RAS components are synthesized in other brain regions like SN and putamen and caudate nuclei (Wright and Harding, 2013; Figure 2).

Renin, responsible for initiating the cascade of angiotensin peptide formation, has been identified in neurons and astrocytes (Bodiga and Bodiga, 2013). Receptors AT1Rs, AT2Rs, ACE, ACE2, and MasRs are found on the cell surface of neurons, endothelial cells, astrocytes, and microglia (Labandeira-Garcia et al., 2017; Jackson et al., 2018) (Table 1 and Figure 2). They have been identified in the mitochondria and cell nuclei (Bodiga and Bodiga, 2013).

**TABLE 1:** Expression of the RAS in neurovascular cells expressed in FPKM.

Gene/protein name	Endothelial cells	Astrocytes	Neurons	Oligodendrocyte	Microglia
AGTR1	0.812 ± 0.638	0.110 ± 0.006	0.555 ± 0.0432	0.1 ± 0.0	0.1 ± 0.0
AGTR2	0.1 ± 0.0	0.1 ± 0.0	0.1 ± 0.0	0.1 ± 0.0	0.1 ± 0.0
ACE	0.416 ± 0.018	0.102 ± 0.002	0.1 ± 0.0	0.1 ± 0.0	0.1 ± 0.0
ACE2	0.1 ± 0.0	0.1 ± 0.0	0.1 ± 0.0	0.1 ± 0.0	0.1 ± 0.0
MasR	0.1	0.1	0.1	0.1	0.1
AGT	1.42 ± 0.11	79.51 ± 13.57	3.3807	1.55 ± 0.19	0.15 ± 0.05

*AGTR1, angiotensin type I receptor; AGTR2, angiotensin type 2 receptor; ACE, angiotensin-converting enzyme; ACE2, angiotensin-converting enzyme 2; MasR, Mas receptor; AGT, angiotensinogen; RAS, renin–angiotensin system.Source: Brain RNA-seq Database. https://www.brainrnaseq.org/.*

The role of the central RAS was first associated with the circulating RAS, e.g., the central control of blood pressure and sodium and water homeostasis, in circumventricular organs lacking the blood–brain barrier (von Bohlen und Halbach and Albrecht, 2006; Phillips and de Oliveira, 2008). Over the last two decades, every component of the classical RAS has been identified in different brain areas near the blood–brain barrier. The brain RAS might be involved in other functions and neurodegenerative processes (Saab et al., 2007; Saavedra, 2012; Goldstein et al., 2016). The components of a local RAS are expressed in the nigrostriatal dopaminergic circuit, and their activation enhances dopaminergic cell vulnerability, inducing dopaminergic cell death via NADPH-derived ROS activation (Meffert et al., 1996; Stroth et al., 1998; Valenzuela et al., 2010; Labandeira-Garcia et al., 2013; Labandeira-García et al., 2014; Zawada et al., 2015; Garrido-Gil et al., 2017). Several studies have shown the nuclear localization (Villar-Cheda et al., 2017; Costa-Besada et al., 2018) and mitochondrial expression (Valenzuela et al., 2016; Garrido-Gil et al., 2017; Costa-Besada et al., 2018) of the different components of RAS in dopaminergic neurons called intracellular or intracrine RAS (iRAS; Grobe et al., 2008; Labandeira-Garcia et al., 2020).

## Effects of Renin–Angiotensin System on Dopaminergic Neurotransmission Pathways

Angiotensin II has been reported to induce DA release in the brain after infusion, and also in dopaminergic cell cultures (Labandeira-Garcia et al., 2013). Angiotensin II perfusion raised the DA level nearly two-fold (Mendelsohn et al., 1993) and was antagonized by the AT1R antagonist losartan (Brown et al., 1996). Furthermore, acute or chronic administration of losartan alone reduced DA synthesis (Dwoskin et al., 1992), suggesting a tonic constitutive effect of Ang II on dopaminergic synapses. Furthermore, chronic administration of candesartan markedly increased DA D1 receptor and decreased DA D2 receptor expression in normal rats (Dominguez-Meijide et al., 2014; Labandeira-Garcia et al., 2020). One study reported the effect of chronic ACE inhibition on striatal DA content and release, using *in vivo* microdialysis in awake, freely moving rats (Jenkins et al., 1997). One week of perindopril treatment largely increased the DA level compared with the untreated control group. The non-peptide compound 21, a selective AT2R agonist, reduced DA synthesis as well (Mertens et al., 2010). These results suggest that Ang II modulates DA synthesis via AT1 and AT2 receptors, which may have opposite effects. Furthermore, Ang II may also regulate postsynaptic dopaminergic receptor level directly or indirectly, affecting the synaptic DA level.

Reserpine-induced DA depletion upregulated the AT1 and AT2 receptors and NADPH oxidase proteins (Labandeira-Garcia et al., 2013). Likewise, 6-OHDA administration induced overexpression of AT1 and AT2 receptors proteins and of NADPH oxidase proteins, which decreased by levodopa (L-DOPA) administration (Labandeira-Garcia et al., 2013). Furthermore, D1 knock-out mice had increased AT1 receptor protein expression in the SNpc and corpus striatum and decreased ACE activity and Ang I, Ang II, and Ang III peptide levels (Villar-Cheda et al., 2010). Conversely, mice overexpressing D2 receptors showed AT2 receptor downregulation (Villar-Cheda et al., 2010). All in all, dopaminergic neurotransmission and the AT1 and AT2 receptor proteins are somehow connected, though the responsible mechanism is not clear. Many studies have pointed out that AT1 and D1 receptors form heterodimeric signaling complexes (Khan et al., 2008; Li et al., 2012). The AT1 receptor may counteract D1 receptor activation, so losartan may induce D1 receptor activation (Li et al., 2012). Also, DA may induce AT1 receptor internalization, which is line with the previously discussed results (Villar-Cheda et al., 2014), and Ang II may induce D1 receptor internalization, reciprocally (Zawada et al., 2015). Interestingly, losartan activates the D1 receptor regardless of DA presence (Scott et al., 2011). The AT1 receptors also dimerize with adenosine A2A receptors (Oliveira et al., 2017), which are major regulators of dopaminergic neurotransmission (Perez-Lloret and Merello, 2014).

These findings suggest sophisticated feedback between DA and Ang II. Accordingly, AT1 receptor activation induces DA synthesis (Mendelsohn et al., 1993; Labandeira-Garcia et al., 2013; Perez-Lloret et al., 2017), which would be inhibited by the AT2 receptor (Brown et al., 1996), and reduces D1 receptor activation. Overall, blockade of the AT1R would facilitate D1-dependent pathways, independently of the DA synaptic level.

In animal models, DA depletion induces compensatory overactivation of the local RAS, which primes microglial responses and neuron vulnerability, activating NADPH oxidase (Labandeira-Garcia et al., 2013). In the corpus striatum and SN, depletion of DA with reserpine upregulated the AT1R, AT2R, and NADPH subunit p47 (phox), all of which decreased as DA function was restored (Villar-Cheda et al., 2010). Neurotoxin-induced loss of dopaminergic neurons is amplified by local Ang II via ATR1 and NADPH complex activation (Villar-Cheda et al., 2010).

In conditions like PD with a sustained dopaminergic deficit, nigrostriatal RAS overactivation is expected to have proinflammatory and degenerative effects (Ruiz-Ortega et al., 2001). Overexpression of Ang II would activate the NADPH oxidase complex, which is responsible for the cell production of ROS (Qin et al., 2004). A high Ang II level has been suggested to exacerbate dopaminergic cell death via AT1R overstimulation and may play a synergistic role in PD pathogenesis and progression (Rodriguez-Pallares et al., 2008; Labandeira-García et al., 2014). Neuronal death has been associated with NADPH oxidase complex activation and ROS production (Joglar et al., 2009). Ang II also acts on microglia, where NADPH oxidase activation produces high concentrations of ROS, which are released extracellularly and affect neurons. Ang II also produces a low level of microglial intracellular ROS, which acts as a second messenger in several microglial signaling pathways involved in the inflammatory response (Babior, 2004; Qin et al., 2004).

In addition to cell death by NADPH oxidase-dependent ROS production (Fazeli et al., 2012), AT1 receptor activation also results in microglia cell degeneration involving other mechanisms like hampering iron homeostasis and releasing proinflammatory interleukins like the tumor necrosis factor alpha (TNF-α; Borrajo et al., 2014; Garcia-Garrote et al., 2019; Garrido-Gil et al., 2013). In sum, the hyperactivation of the local RAS exacerbates the inflammatory microglial response, oxidative stress, and dopaminergic degeneration, all of which are inhibited by Ang receptor blockers and inhibitors of the ACEs (Labandeira-Garcia et al., 2013).

The role of AT2 receptors in dopaminergic pathways has been less studied. The abundance of AT2R in fetal tissues is consistent with high cell proliferation, maturation, apoptosis, and regeneration activity (Garcia-Garrote et al., 2019). Treatment of neurospheres with 100 nM of Ang II during cell differentiation induced a striking four-fold increase in dopaminergic neuron generation (Rodriguez-Pallares et al., 2004). Neuronal differentiation and growth have been reported to follow AT2R activation (Gallo-Payet et al., 2011). The activation of AT2 receptors promotes neuronal growth associated with cytoskeleton rearrangements critical to induce neurite elongation (Laflamme et al., 1996; Meffert et al., 1996). Neuronal growth is also associated with an increase in mature neural cell markers like βIII-tubulin and microtubule-associated proteins (MAPs) like MAP2c (Laflamme et al., 1996; Stroth et al., 1998). Different mechanisms have been put forward to explain how AT2 activation causes neuronal differentiation and growth. Angiotensin promotes neurosphere differentiation and overexpression of methyl methanesulfonate sensitive 2 (MMS2), a neuronal growth factor involved in DNA repair (Mogi et al., 2006). Once activated, AT2 receptors form a complex with the AT2 receptor-interacting protein (ATIP protein) and translocate to the nucleus where MMS2 transactivation takes place (Nouet et al., 2004; Horiuchi and Mogi, 2011). Knockdown of the MMS2 gene by small interfering RNA (siRNA) reduced the number of neurospheres with loss of sphere formation (Mogi et al., 2006). The angiotensin II type 1 receptor blocker, valsartan, enhanced neurosphere differentiation and MMS2 induction, while the AT2R antagonist PD123319 caused inhibition (Mogi et al., 2006). The overall evidence supports a neuroprotective role for the AT2R receptor.

Stimulation of peroxisome proliferator-activated receptor gamma (PPAR-γ) downregulates AT1 receptors in vascular smooth muscle cells (VSMCs; Takeda et al., 2000). Angiotensin II induces PPAR-γ activation in PC12W cells via AT2R activation (Zhao et al., 2005). The nuclear PPAR-γ receptor controls lipid and glucose metabolism, energy homeostasis, and adipocyte and macrophage differentiation and has a neuroprotective function (Guimond and Gallo-Payet, 2012). The PPAR-γ was reported to participate in downregulating several inflammatory cytokines and inhibiting inflammation (Delerive et al., 2001). These lines of evidence suggest that neuroprotection via the AT2 receptor might involve PPAR-γ activation.

Many authors accept that AT2R and AT1R have antagonistic effects. Ang II protected dopaminergic neurons *in vitro* against MPP+ toxicity only in the presence of the AT1R antagonist losartan, while losartan did not affect MPP+ toxicity (Grammatopoulos et al., 2007). This suggests that Ang II neuroprotection involves the AT2R. Activation of the AT2R promotes cell growth and differentiation involving the MMS2 and PPAR-γ pathways. Angiotensin receptor blockers (ARBs) activate PPAR-γ receptors. Then, ARBs may act not as AT1 inhibitors but as AT2 agonists, activating the AT2–PPAR axis.

### Potential Clinical Applications of Renin–Angiotensin System Modulation in Parkinson’s Disease

Dopaminergic therapy is the most accepted symptomatic treatment in PD, targeting dopaminergic neuronal loss in the SN and the resulting striatum dopamine depletion at motor symptom onset. However, it is not devoid of long-term secondary effects. An evidence-based alternative treatment targeting RAS modulation could be used for PD symptom management upon its participation in SN DA cell survival and function (Perez-Lloret et al., 2017).

One of the long-term L-DOPA treatment consequences is L-DOPA-induced dyskinesias (LIDs), bound to neuroinflammation and angiogenesis, due to overstimulation of the D1-dependent so-called “direct” pathway, causing abnormal choreoathetotic movements (Fabbrini et al., 2007; Muñoz et al., 2014). Most L-DOPA-treated PD patients are eventually affected by LIDs and become disabled (Olanow et al., 2013). One study tested the AT1R blocker candesartan on (s.c. injection) LIDs in 6-OHDA-lesioned rats. Overexpression of vascular endothelial growth factor (VEGF) and the neuroinflammatory IL-1β were used in the study as possible LID predictors, linked to neuroinflammation and angiogenesis (Muñoz et al., 2014). Dyskinesia was reduced in the candesartan-treated rats compared with the L-DOPA alone-treated animals (control group). Candesartan treatment attenuated limb and orolingual movements without affecting axial LIDs or interfering with antiparkinsonian L-DOPA efficacy. The L-DOPA-lesioned rats had increased n VEGF and IL-1β expression in the SN and corpus striatum areas than had candesartan-treated non-dyskinetic rats (Muñoz et al., 2014; Perez-Lloret et al., 2017). Angiotensin II addition to primary mesencephalic cultures or intracerebroventricularly injected in rats enhanced VEGF expression, as found postmortem, and its effects were blocked by candesartan (Muñoz et al., 2014; Perez-Lloret et al., 2017).

The effects on motor fluctuations of the ACE inhibitor perindopril were examined in a double-blind, placebo-controlled, crossover pilot study in seven subjects with PD. The subjects were diagnosed with moderately severe PD according to the Hoehn and Yahr scale. Patients received either placebo or perindopril for 4 weeks while under L-DOPA treatment. The clinical response to L-DOPA test dose, dyskinesia, “on” and “off” state daily life activities, and motor fluctuations was evaluated. Perindopril improved the motor response to L-DOPA treatment, increasing the overall amplitude of response with a faster onset of action and a reduction in the “on” phase dyskinesia peak, although perindopril resembled a dopaminergic transmission modulator in seven subjects only (Reardon et al., 2000).

The nigrostriatal local RAS has a promising potential for the symptomatic management of PD as of its participation in regulating dopaminergic pathways and inflammatory responses (Perez-Lloret et al., 2017). The study by Reardon and colleagues sets a precedent for further clinical studies, as for the positive response to perindopril, in six out of seven subjects studied (Reardon et al., 2000). Additional studies should be conducted to have a better comprehension of the cellular structures involved in the effects observed and confirm the clinical benefits of these drugs, if so.

## Conclusion and Future Directions

PD is characterized by the loss of dopaminergic synapses in the nigrostriatal pathway, which may actually precede the neurodegenerative process. The RAS has a modulatory role on the dopaminergic synaptic function and affects the survival of SNpc neurons (Perez-Lloret et al., 2017), highlighting its potential as a target of new symptomatic and disease-modifying therapies. Experimental evidence suggests that a low DA level stimulates angiotensin II synthesis and release from astrocytes and vice versa, via the AT1 receptor, while AT2R activation might counterbalance, reducing the DA level. This might help to restore the nigrostriatal dopaminergic tone in PD. In the long term, RAS overactivation leads to excessive ROS production, microglia activation, and cell death. Therefore, AT1R blockade or AT2R stimulation might contribute to reducing the oxidative stress burden in the SN during the parkinsonianstate or even before.

A negative allosteric relationship has been established between AT1 and D1 receptors. Blockade of AT1 activates the D1 receptor, even in the absence of L-DOPA. In theory, the D1-dependent so-called “direct pathway” would be activated, restoring the balance with the D2-dependent “indirect pathway” overactivation, lost after dopaminergic loss (Grace, 2008). The clinical implications of this hypothesis are straightforward and have been partially tested. In a small, double-blind, crossover trial, a 2-week treatment with 2 mg of perindopril not only increased L-DOPA antiparkinsonian efficacy, which would be expectable by the activation of D1 receptors, but showed antidyskinetic effects, as well (Reardon et al., 2000). Studies in rodent models of parkinsonism showed that candesartan reduced LIDs without affecting L-DOPA antiparkinsonian effects. These observations are at odds with the expected pro-dyskinetic effects of the D1-dependent “direct pathway” activation (Grace, 2008). The effects of other RAS molecules on the function of the dopaminergic synapses have been less studied and might be the cause of these challenging observations.

We conclude that modulating dopaminergic neurotransmission with ACE inhibitors or AT1 antagonists might have antiparkinsonian effects, which deserve further research. Studies focusing on perindopril are encouraged, given the accumulated evidence on its antiparkinsonian effects. More research is also needed on the AT2 receptor effects.

## Author Contributions

SP-L, and MO-L: conception and original idea. TK, SP-L, GC, LU, SB, and CM-M: investigation. SP-L, MO-L, and TK: original drafting. MO-L: critical revision and editing. FC: funding acquisition. All authors contributed to the article and approved the submitted version.

## Conflict of Interest

The authors declare that the research was conducted in the absence of any commercial or financial relationships that could be construed as a potential conflict of interest.

## Abbreviations

ACE: angiotensin-converting enzyme
ACE2: angiotensin-converting enzyme 2
ACEi: ACE inhibitor
Ang: angiotensin
ARB: angiotensin receptor blocker
AT1R: angiotensin receptor type 1
AT2R: angiotensin receptor type 2
ATIP: AT2 receptor-interacting protein
ATP: adenosine triphosphate
CHL: cholinergic
CNS: central nervous system
DA: dopamine
GLU: glutamate
GDNF: glial cell line-derived neurotrophic factor
IL-1β: interleukin-1 beta
iRAS: intracellular or intracrine RAS
LBs: Lewy bodies
L-DOPA: levodopa
Leu: leucine
LIDs: levodopa-induced dyskinesias
LNs: Lewy neurites
MasR: Mas receptor
MDS-UPDRS II + III: Movement Disorder Society (MDS)—Unified Parkinson’s Disease Rating Scale (UPDRS)
NADPH: nicotinamide adenine dinucleotide phosphate
NMDA: *N*-methyl -D-aspartate
PD: Parkinson’s disease
PPAR-γ: peroxisome proliferator-activated receptor gamma
RAS: renin–angiotensin system
ROS: reactive oxygen species
SN: substantia nigra
SNpc: substantia nigra pars compacta
TNF-α: tumor necrosis factor alpha
VEGF: vascular endothelial growth factor.
